# NETopathic Inflammation in Chronic Obstructive Pulmonary Disease and Severe Asthma

**DOI:** 10.3389/fimmu.2019.00047

**Published:** 2019-02-05

**Authors:** Mohib Uddin, Henrik Watz, Anna Malmgren, Frauke Pedersen

**Affiliations:** ^1^Respiratory Global Medicines Development, AstraZeneca, Gothenburg, Sweden; ^2^Respiratory, Inflammation and Autoimmunity, IMED Biotech Unit, AstraZeneca, Gothenburg, Sweden; ^3^Pulmonary Research Institute at LungenClinic, Großhansdorf, Germany; ^4^Airway Research Center North (ARCN), German Center for Lung Research (DZL), Großhansdorf, Germany; ^5^LungenClinic, Großhansdorf, Germany

**Keywords:** airway, asthma, COPD, CXCR2, neutrophil extracellular traps (NETs), NETopathic inflammation

## Abstract

Neutrophils play a central role in innate immunity, inflammation, and resolution. Unresolving neutrophilia features as a disrupted inflammatory process in the airways of patients with chronic obstructive pulmonary disease (COPD) and severe asthma. The extent to which this may be linked to disease pathobiology remains obscure and could be further confounded by indication of glucocorticoids or concomitant respiratory infections. The formation of neutrophil extracellular traps (NETs) represents a specialized host defense mechanism that entrap and eliminate invading microbes. NETs are web-like scaffolds of extracellular DNA in complex with histones and neutrophil granular proteins, such as myeloperoxidase and neutrophil elastase. Distinct from apoptosis, NET formation is an active form of cell death that could be triggered by various microbial, inflammatory, and endogenous or exogenous stimuli. NETs are reportedly enriched in neutrophil-dominant refractory lung diseases, such as COPD and severe asthma. Evidence for a pathogenic role for respiratory viruses (e.g., *Rhinovirus*), bacteria (e.g., *Staphylococcus aureus*) and fungi (e.g., *Aspergillus fumigatus*) in NET induction is emerging. Dysregulation of this process may exert localized NET burden and contribute to NETopathic lung inflammation. Disentangling the role of NETs in human health and disease offer unique opportunities for therapeutic modulation. The chemokine CXCR2 receptor regulates neutrophil activation and migration, and small molecule CXCR2 antagonists (e.g., AZD5069, danirixin) have been developed to selectively block neutrophilic inflammatory pathways. NET-stabilizing agents using CXCR2 antagonists are being investigated in proof-of-concept studies in patients with COPD to provide mechanistic insights. Clinical validation of this type could lead to novel therapeutics for multiple CXCR2-related NETopathologies. In this Review, we discuss the emerging role of NETs in the clinicopathobiology of COPD and severe asthma and provide an outlook on how novel NET-stabilizing therapies via CXCR2 blockade could be leveraged to disrupt NETopathic inflammation in disease-specific phenotypes.

## Neutrophils

Optimal regulation of neutrophil-mediated immunity is essential for confining pathogens, resolving infection and inflammation (1). Neutrophils are the most abundant circulating leukocyte and are first responders of the host-protective innate immunity (2). These cells fulfill their sentinel role by safeguarding the host immune homeostasis through maintaining a strict equilibrium of the innate immune and acute inflammatory responses (3). While a robust innate immune response is vitally important for an effective host defense, dysfunction of neutrophils is associated with bystander immune responses in certain treatment-refractory lung diseases (4, 5).

Circulating neutrophils are short-lived innate immune cells and limited by an inherent pathway of apoptosis. Apoptosis is a form of programmed cell death and phagocytic clearance of dying neutrophils by macrophage facilitates the resolution of inflammation (6). Defective neutrophil apoptosis has been reported in the airways of patients with asthma with the degree of dysfunction corresponding to disease severity (7), suggesting derangements in inflammation resolution. Conflicting data exist on the potential role of impaired apoptosis in COPD (8, 9), whereas defects in efferocytosis have been identified in both neutrophilic asthma (10) and COPD (11). Targeting defective cell death pathways in these refractory airway diseases represents an attractive therapeutic strategy.

## Neutrophil Extracellular Traps (NETs)

Neutrophils kill and clear invading pathogens via phagocytosis, degranulation and oxidative burst. There is an increasing recognition that neutrophils can extrude cytosolic and nuclear material via a conserved cell death process distinct to apoptosis and necrosis (12). This active process is commonly referred to as neutrophil extracellular traps (NETs) and was first coined in 2004 by Brinkmann and colleagues as a novel antimicrobial defense system (13). NETs are web-like scaffolds of extracellular DNA in complex with histones and antimicrobial neutrophil granular proteins, such as myeloperoxidase and neutrophil elastase (13). Entrapment of microorganisms by NETs restrict potential pathogen dissemination from the initial site of infection and it is hypothesized that electrostatic interactions govern the specificity of binding between the cationic nature of NETs and the anionic bacterial cell wall (14). Physicochemical perturbations are reported to modulate NET formation, including hypoxia, osmolarity, and extracellular pH shifts (15–19).

**Figure 1:**
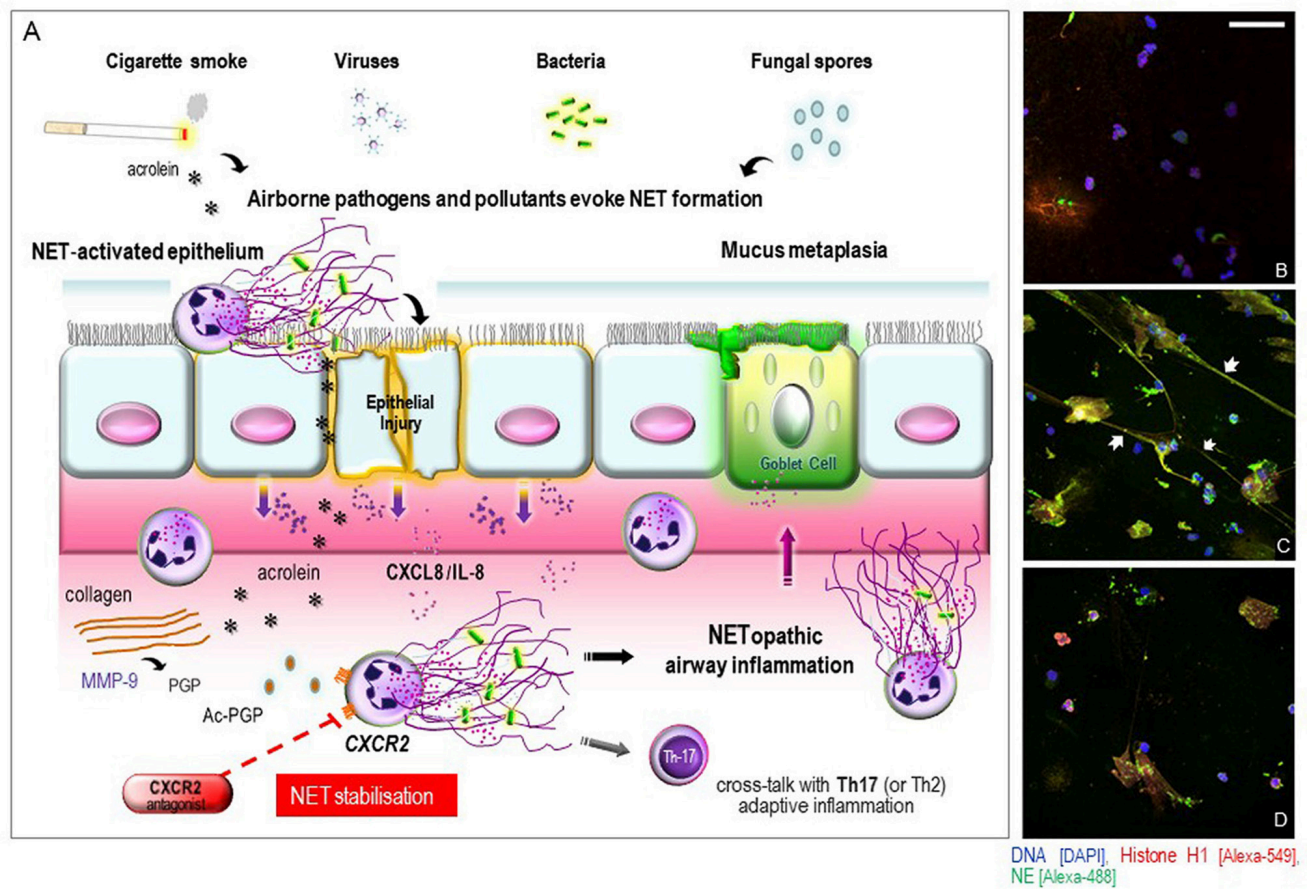
**(A)** schematic illustration of proposed mechanisms of airway epithelial and innate immune cell responses to NET induction in COPD or in the asthma smoking phenotype and the potential influence of CXCR2 signaling. Exposure to airborne pathogens and pollutant stimuli evoke a NET-permissive microenvironment leading to a cycle of airway mucosal inflammation. A dysregulated epithelium releases neutrophil-attracting mediators that can transduce their effects via CXCR2 signaling. The oxidants in cigarette smoke and other reactive components (e.g., acrolein) can also directly trigger NET formation or via acetylation of proteolytically cleaved collagen PGP to yield potent CXCR2-signaling matrikines, attracting neutrophils into the airways to further perpetuate NETopathic inflammation. NETs may also contribute to mucus-hypersecretion by submucosal glands via induction of respiratory mucins. Additionally, defective NET production can induce Th2 and Th17 adaptive immune responses which may further contribute to the disease pathomechanism. Our hypothesis is that selective CXCR2 antagonists [e.g., AZD5069] could essentially block CXCR2-stimulated NET induction, and may thereby represent a potential NET-stabilizing agent capable of disrupting NETopathic airway inflammation in NET-rich COPD or in the asthma smoking phenotype. **(B–D)** CXCR2 receptor antagonism stabilizes heightened NET formation in COPD *ex vivo*. Representative merged 3D confocal-fluorescence images of the NETs formed in COPD-derived peripheral blood neutrophils in response to stimulation with autologous COPD sputum supernatant from the same consenting patient. All specimens were fixed in phosphate buffered 4% paraformaldehyde. Immunofluorescence staining for detection of histone H1 (*green*), neutrophil elastase (*red*) and nuclear DNA with 4,6-diamidino-2-phenylindole (DAPI) (*blue*) was performed as described previously (30). Representative visual fields were acquired using a confocal laser scanning microscope (Leica microsystems TCS SP5) and captured by applying identical magnification, laser intensities, and detector settings. **(B)** unstimulated blood neutrophils with DAPI staining showing nuclear DNA; **(C)** blood neutrophils stimulated with autologous COPD sputum supernatant *ex vivo* with merges of the three colocalized images for histone H1, neutrophil elastase, and DAPI staining indicating projection of NET formation (arrowheads); **(D)** blood neutrophils stimulated with autologous COPD sputum supernatant after pretreatment with 100 μM AZD5069 *ex vivo*, with merges of the three color images indicating stabilized NET formation. Scale bar 50 μm.

Though NETs are considered an essential part of neutrophil-mediated immunity, they have also been incriminated in NET-based immunopathology (20, 21). In a sense, NETs represent a “double-edged sword” in innate immunity (22), heavily dependent on maintaining a tight equilibrium between protective and detrimental immune responses. Aberrant NET production in the circulation and affected tissues have been reported in patients with cystic fibrosis and ARDS (23–25)—airway diseases typified by neutrophilic mucosal inflammation. Persistence of exaggerated NET formation may induce collateral damage to the airway tissues. For instance, NETs can directly trigger epithelial cell death (26) or when excessive, may impair lung epithelial barrier function during respiratory viral infection *in vivo* (27). Murine studies have demonstrated a role for NETs in inducing airway mucus hypersecretion (28). Furthermore, emerging translational studies lend further support to the assertion that NET burden could have a pathobiological role in treatment-refractory airways diseases. We (29, 30) and others (31–33), have recently provided the first evidence for induction of NET formation in neutrophils derived from COPD and severe asthma patients, suggesting that this process may also influence airway immunopathology (“lung NETopathy”) in these diseases (Table 1). A schematic representation of the proposed mechanism involving NETs in mediating airway NETopathic inflammation in NET-rich COPD and in the asthma smoking phenotype is depicted on Figure 1A.

**Table 1:** Overview of translational evidence of NET formation in patients with COPD and asthma.

**Patient population (*n*)**	**Key translational findings using patient-derived samples**	**References**
**COPD** (*n* = 6)	Increased NET production following LPS stimulation in peripheral blood-derived neutrophils from a small cohort of patients with stable COPD compared with healthy controls	(41)
**COPD** (*n* = 16)	Increased levels of NETs present in induced sputum samples from exacerbated COPD patients	(31)
**COPD** (*n* = 23)	Enhanced NET formation in induced sputum from stable COPD patients which correlated positively with airway neutrophil numbers and high concentrations of extracellular DNA	(29)
**COPD** (*n* = 44)	Abundant presence of sterile NETs in the sputum of patients with stable and exacerbated COPD that correlated with degree of airflow limitation [FEV_1_] and disease severity	(32)
**COPD** (*n* = 44)	Sputum NETs and airway neutrophils were inversely proportional to lung function and symptoms. Expression of PAD4 mRNA was upregulated in neutrophilic COPD	(42)
**COPD** (*n* = 99)	Increased sputum NET levels were associated with COPD severity (GOLD criteria), non-eosinophilic COPD exacerbations, reduced bacterial diversity and increased *Haemophilus* species	(45)
**COPD** (*n* = 12)	Enhanced NET induction in autologous blood and sputum neutrophils from COPD patients, this response was stabilized using the CXCR2 antagonist, AZD5069. This is the first mechanistic study to show an association specifically between CXCR2 signaling and NET stabilization in COPD *ex vivo* (Figures 1B–D)	(30)
**Asthma** (*n* = 20)	Accumulation of NETs and eosinophil extracellular traps (EETs) present in the bronchial biopsies of atopic asthmatics	(106)
**Asthma** (*n* = 94)	Raised levels of NETs detected in induced sputum derived from neutrophilic asthmatic relative to non-neutrophilic asthmatics that were inversely correlated to lung function and disease control	(42)
**Asthma** (*n* = 68)	Peripheral blood-derived neutrophils from severe asthmatics displayed greater NET production after CXCL8/IL-8 stimulation relative to cells from non-severe patients. These NETs induced airway epithelial damage and stimulated release of endogenous epithelial CXCL8/IL-8 production.	(33)
**Asthma** (*n* = 23)	Increased release of dsDNA following rhinovirus infection *in vivo* that was related to type-2 cytokine induction and exacerbation severity in asthmatics	(82)

## NETopathic Inflammation in COPD

COPD is projected to become the third leading cause of death world-wide and is a major cause of mortality in adults (34). It is characterized by airflow limitation that is poorly reversible caused by narrowing of the small airways combined with emphysematous destruction (34). COPD is heterogeneous and encompasses a clinical spectrum of disease-specific phenotypes (35). Chronic exposure to cigarette smoke has been recognized to contribute to COPD pathogenesis (34), where it can elicit neutrophil retention within the airways (36). In general, the degree of neutrophilia correlates with COPD severity (37, 38), exacerbations (39), disease progression (40), and GC resistance (35).

Two independent translational studies in 2015, by Pedersen et al. (29) and Grabcanovic-Musija et al. (32) first observed NET formation in sputum from both stable and exacerbated COPD patients using confocal fluorescent and electron microscopy, respectively. These findings suggested that NET formation may be a feature of a NET-rich COPD phenotype. NET production has been shown to occur in peripheral blood-derived neutrophils from a small cohort of patients with stable COPD (41). Elevated sputum NETs are also associated with lung function and COPD symptoms and peptidylarginine deiminase 4 (PAD4) gene expression was found to be upregulated in neutrophilic COPD relative to non-neutrophilic patients (42). The increase in PAD4 expression at the protein level has previously been reported in the lungs of COPD patients (43), and it is believed that activation of PAD4 is an important regulator of NET formation (44). More recently, Dicker and colleagues reported increased NET formation in the airways of patients of COPD that was associated with disease severity (45). They went onto demonstrate a relationship between sputum-enriched NETs and non-eosinophilic COPD exacerbations and reduced bacterial diversity accompanied by an abundance of *Haemophilus* species. Functionally, the phagocytic capacity of neutrophils to engulf bacteria *ex vivo* was impaired in cells derived from patients with high sputum NET complexes, supporting their potential role in microbial dysbiosis in COPD (45).

## CXCR2 Signaling in COPD

CXCR2 is a G-protein-coupled receptor that binds human CXC chemokine ligands, such as CXCL8/IL-8, CXCL1/GRO-α, and CXCL5/epithelial neutrophil-activating peptide-78 (ENA-78) with high affinity (46, 47). CXCL8/IL-8 is considered to be the predominant neutrophil-attracting chemokine in COPD airway secretions, accounting for the trafficking of approximately one-third of neutrophilic infiltrates in sputum (48). Additional CXCR2 ligands, such as CXCL1/GRO-α and CXCL5/ENA-78 are elevated in COPD sputa, airway fluids, and bronchial tissues (38, 49, 50). CXCR2 is upregulated in exacerbations of COPD where its expression co-localizes with accumulation of airway mucosal neutrophils (49). Recent clinical studies with the CXCR2 antagonist MK-7123 (SCH527123) in patients with COPD have shown a significant neutrophil-lowering effect leading to improvements in FEV_1_ and a reduction in exacerbations in active smokers compared to placebo (51). However, a dramatic fall in circulating neutrophils (neutropenia) was noted in a large proportion of COPD patients, raising concerns about undesirable immunosuppression. Avoidance of off-target effects are the major safety concerns to address, however, there has been scientific debate as to whether this could be mitigated by local delivery of inhalable CXCR2 antagonists directly to the lungs. From an efficacy perspective, it remains to be determined whether the observed anti-CXCR2 effects on improved lung function in that study were associated with NET stabilization within the COPD airways.

As further clinical trial data with CXCR2 antagonists emerge in NET-rich lung diseases, it will be important to dissect how well the steroid-resistant phenotypes respond to these neutrophil-trafficking CXCR2 inhibitors. Further mechanistic insights into CXCR2-mediated signaling is emerging from studies involving extracellular matrix (ECM)-derived fragments, such as the pro-neutrophilic matrikine, proline-glycine-proline (PGP). This tripeptide has structural homology to ELR+ chemokines and can chemotactically recruit neutrophils via CXCR2 receptors (52). Levels of PGP are markedly raised in the airways of patients with COPD (52, 53) which can occur in response to cigarette smoke (54). This airway irritant is also able to directly induce NET production (55), through its bioactive constituents, such as the reactive aldehyde and tussive agent acrolein (56). Acrolein can induce release of PGP fragments, including acetyl-proline-glycine-proline [AcPGP] (57) that function as potent CXCR2 signaling ligands. AcPGP has been utilized as a potential biomarker in a recent clinical study involving COPD patients with chronic bronchitis (58). Expanding the concept of PGP-CXCR2 crosstalk in COPD, we propose it is pharmacologically plausible that PGP could also trigger NET formation during COPD-related airway inflammation. Future studies need to establish whether PGP can directly contribute to lung NETopathologies in a CXCR2-dependent manner.

Interestingly, emerging evidence also implicates CXCR2 signaling in regulating NET production in COPD neutrophils. Selective anti-CXCR2 blockade using AZD5069 limited NET formation in autologous blood and sputum neutrophils derived from the same COPD patients *ex vivo* (30), representing the first reported evidence that a CXCR2 antagonist can stabilize COPD-derived NET formation (Figures 1B–D). This unique profile of CXCR2 dependence in regulating COPD-related NETopathic inflammation, and its consequent antagonism, differentiates it from the conventional neutrophil-trafficking functions. Applying this approach clinically, it is noteworthy that CXCR2 antagonists are now being trialed to evaluate their modulatory effects on sputum NET production in patients with COPD [ClinicalTrials.gov-Identifier: NCT03250689]. If successful, this could introduce an emerging field of therapeutic research for “NET stabilizers” in a range of CXCR2-related NETopathologies, including NET-rich COPD.

## Pulmonary Pathogen-initiated NETopathic Inflammation

Respiratory viral infections, such as respiratory syncytial virus (RSV), rhinovirus and *influenza virus A*, are associated with exacerbations of COPD (59) and can induce NET formation (60–62). NETs have been ascribed an anti-viral immune role (63, 64), involving molecular interactions between NET-derived bactericidal/permeability-increasing protein and the fusion (F) protein on the viral surface (65). However, an aberrant NETopathic response can be damaging to the host when excessive. This is consistent with murine studies demonstrating that viral-induced NET perturbance can elicit acute lung injury and airway inflammatory responses following challenges with *influenza virus A* and *parainfluenza virus* type I (Sendai virus) in experimental models of pneumonitis and asthma *in vivo*, respectively (27, 66).

COPD patients are highly susceptible to recurrent bacterial infections which often occur after encountering respiratory viral infections (67). Chronic airway bacterial colonization with the pathogenic organisms, *Haemophilus influenzae* and *Streptococcus pneumoniae* are strongly associated with recurrent COPD exacerbations (68, 69). The pneumonia-causing pathogen, *Staphylococcus aureus* (*S. aureus*), has also been detected in the airways of COPD patients (70, 71), notably in much older populations (72). This is particularly important because *S. aureus* can directly trigger NET formation (73), and might thus influence the cycle of pathogen–NETopathic inflammation in COPD. Despite there being high levels of NETs in the COPD airways, there is a deficiency in effective pathogen clearance. Reports suggest that community-acquired pneumonia (CAP) is more common in patients with COPD relative to the adult general population and is higher in the winter period (67). Circulatory NETs have recently been shown to be associated with 30-day all-cause mortality in a cohort of 310 patients with CAP of whom 17.4% had COPD as a comorbidity (74). These findings are suggestive of an innate immunity conundrum we refer to as “the NET paradox”; if NET-forming neutrophils are committed to the eradication of invading microbes, then why do respiratory pathogens frequently colonize and induce pathogenicity in the CAP airways? Recent findings indicate that certain respiratory pathogens have developed effective mechanisms to evade NET-mediated entrapment and killing [reviewed in (75)]. This immune evasion strategy deployed by respiratory pathogens could explain the apparent NET paradox in pathogen-colonized airways in these patients, and might be an important factor in determining the outcome of CAP infections.

In asthma, respiratory viral infections represent acute pathobiological events that can precipitate exacerbations (76, 77). Rhinovirus-induced respiratory infections elicit rapid recruitment of neutrophils in the asthmatic airways with a concomitant increase in CXCL8/IL-8 levels (78, 79) and degree of airway responsiveness (80). It has been shown that CXCR2 signaling is essential for rhinovirus-induced neutrophilic airway inflammation and development of airway hyperresponsiveness in murine model *in vivo* (81). Recent work by Toussaint et al. have shown that NETs cross-talk with viral-provoked immune responses in the respiratory tract, thereby driving type-2 allergic airway inflammatory responses (82, 83). Elevated levels of host double stranded DNA (dsDNA) were observed in the upper asthmatic airways elicited by rhinovirus infection that correlated with subsequent type-2 immune-mediated asthma exacerbation severity. The study recapitulated the pathobiological features that mimic RV-induced asthma exacerbation using a house dust mite (HDM)-allergen-sensitized mouse model *in vivo*. Depletion of dsDNA production using DNase was found to constrain RV-induced exacerbation and hyper-responsiveness after HDM allergen challenge (82), in agreement with reports of lung function improvements in another pre-clinical model of asthma (84). Further interrogation of the role and regulation of viral-induced NETopathy with synergistic interaction following allergen exposure in triggering asthma exacerbations are warranted.

In addition to respiratory viral and bacterial infections, airborne transmission of fungi is associated with a subphenotype of asthma denoted as severe asthma with fungal sensitization (SAFS) (85). Sensitization to *Aspergillus fumigatus* and consequent airway neutrophilic inflammation correlate with asthma severity and reduced lung function (86). An essential role for NETs in the local confinement of *A. fumigatus* has been reported *in vitro* (87) and *in vivo* (88). It remains to be seen whether fungal-neutrophil interactions could uncover specific pathological roles in driving NETopathic airway inflammation in severe asthma.

## NETopathic Inflammation in Asthma

Severe asthma is distinguished from milder forms of the disease by distinct underlying endotypes (89, 90). Neutrophilic predominant asthmatic inflammation differs from Th2-high eosinophilic inflammation, thus often being associated with Th2-low asthma (T2-low) (91–93). The presence of viable neutrophils in the airways often correlates with poorer clinical outcome, disease severity and response to GC therapies (7, 94–100). Translational evidence incriminating neutrophils in the pathobiology of severe asthma have provided a rationale for exploring therapeutic agents that selectively block inflammatory pathways (4, 101–103). However, the precise nature of neutrophilic asthmatic inflammation remains contentious (104, 105).

In asthma, both NETs and eosinophil extracellular traps (EETs) have been detected in bronchial biopsies from atopic patients, although levels were not different between pre- and post-allergen challenge (106). Increased NETs and their components, namely extracellular DNA and LL-37, as well as CXCL8/IL-8 in induced sputum were significantly higher in neutrophilic asthmatics relative to the non-neutrophilic cohort (42). This was inversely correlated with lung function and asthma control, suggestive of a potential link between airway NETopathy and worst asthma outcomes. Further, peripheral blood-derived neutrophils from severe asthmatics displayed greater NET production than did cells from non-severe patients after CXCL8/IL-8 stimulation *ex vivo* (33). Excessive NET load has been theorized as a potential driver for disordered mucosal epithelium in relation to asthma severity. *Ex vivo* stimulation of airway epithelial cells with NETs can markedly induce endogenous CXCL8/IL-8 production (33, 107), creating a possible self-amplifying cycle of airway inflammation, consistent with reports of a mucosal epithelial-neutrophil interplay in severe asthma (108). In addition, recent findings show that a subset of severe asthmatics has NETopathic inflammation mediated by neutrophil cytoplasts which may skew the immune responses toward Th17 adaptive inflammation (109, 110). Augmented NET formation has also been reported in eosinophilic granulomatosis with polyangiitis (EGPA; traditionally termed Churg–Strauss), an asthma plus syndrome characterized by eosinophilic inflammation and paranasal sinusitis (111). Circulatory NETs have been shown to positively correlate with anti-lactoferrin-specific antibody titers in patients with EGPA in another study (112). Together, these data indicate that defective NET formation might underpin some aspects of airway inflammation in more severe asthma.

## CXCR2 Signaling in Severe Asthma

Elevated expression levels of CXC chemokines (CXCL1/GRO-α, CXCL5/ENA-78, or CXCL8/IL-8) associate with increased neutrophil infiltrates in the asthmatic bronchial mucosa, airway wall, and sputum (113–117). Anti-CXCR2 blockade can negate multiple features of pulmonary neutrophilic inflammation *in vivo*, supporting a pro-inflammatory role for CXCR2 signaling (118). AZD5069 is a selective CXCR2 antagonist developed by AstraZeneca that blocks neutrophil trafficking without hindering neutrophil effector responses of host defense (119, 120). Recently, AZD5069 has been shown to diminish CXCR2-mediated neutrophil infiltrates in spontaneous sputum from bronchiectasis patients (69% inhibition) (121), as well as pronounced reduction in induced sputum and bronchial biopsies from patients with more severe asthma (90% inhibition) (122). Treatment with another oral CXCR2 antagonist (MK-7123) was shown to attenuate sputum neutrophilia by 36% in patients with severe asthma (*n* = 34), yet had no clinical effect on asthma outcomes (123). Despite the observed neutrophil infiltrate lowering effects in severe asthma, results from trials of orally-active CXCR2 antagonists have not shown any clinical benefits to date.

More recently, a larger (6 months/*n* = 640) double-blind randomized phase 2b study has been conducted using three different doses of AZD5069 in patients with uncontrolled persistent asthma (102). AZD5069 induced a dose-related and sustained reduction in circulating neutrophils, whereas no impact was observed on the clinical outcomes in that study which had an overall low severe exacerbation rate, leaving uncertainties about the pathomechanism of CXCR2-mediated neutrophil recruitment in severe asthma. Targeting well-defined endotypes of neutrophilic non-eosinophilic/T2-low asthma using a biomarker-directed approach has recently been proposed (101, 103). In this context, the exclusion of patients who were current smokers or ex-smokers (smoking history of ≥ 20 pack years) in this clinical study (102), meant that it was not possible to determine the therapeutic potential of CXCR2 antagonists in smokers with asthma. Bearing in mind that nearly a quarter of adult asthma patients are current cigarette smokers in the “real world” (124), chronic exposure to tobacco smoke can modify the airway inflammatory profile (whilst reducing fractional exhaled nitric oxide [FeNO] levels) and worsen clinical outcomes in smokers with more severe asthma (125, 126). Data from the recent U-BIOPRED cluster analyses highlight that smoking pack year history differentially induces a pro-inflammatory signature profile (but not Th2-associated) in sputum of patients with severe asthma (127). As noted, cigarette smoke exposure leads to induction of NET formation (55) in association with CXCL8/IL-8 upregulation in the airway mucosa of smoking asthmatics (128), an inflammatory response that is insensitive to systemic GC therapy (129). This could lead to a feed-forward process of smoke-triggered CXCR2-dependent neutrophil accumulation, thereby maintaining the chronicity of NETopathic inflammation in the airways of the asthma smoking phenotype (Figure 1A).

## Concluding Remarks

In just a decade, the identification of NETs has reshaped the paradigm of neutrophil-mediated innate immunity. Disentangling the function of NET biology in human health and disease is ongoing. It is now recognized that NET formation is an effective antimicrobial defense system, yet its dysregulation may impart bystander consequences and airway NETopathic inflammation. Despite considerable research efforts, important questions remain unanswered in the context of treatment-refractory lung diseases. For instance, how the upstream cues consequent to airborne insults (e.g., respiratory viruses, cigarette smoke, fungal exposure) are integrated during induction of NET perturbance (Figure 1A) and whether they operate concurrently and/or redundantly in COPD or the smoking asthma phenotype. Furthermore, there is an apparent NET paradox of heightened infective susceptibility to respiratory pathogens in the face of concomitant NETopathic airway inflammation, and whether there is a preferential deployment of NET-forming subsets of neutrophils? Whether and how NET formation contributes to the pathogenesis of COPD and in the smoking asthma phenotype, or stabilizing this process may be an efficacious therapeutic strategy is unknown. It is important to consider that NETs are vital to host defense and that therapeutic intervention could cautiously be aimed at stabilizing the aggressive potential of NETs to homeostatic control rather than completely neutralizing this process as an ideal outcome. In any case, in our opinion, it seems reasonable that stabilizing airway NET burden might provide a rationale for disrupting this process in NET-rich COPD or in the smoking asthma phenotype. These studies await clinical validation in phenotypically-defined patient populations. With no curative therapy currently available for these fatal lung diseases, we envisage that disentangling the pathways underpinning NETopathic inflammation could lead to the development of innovative therapeutics for disease-specific phenotypes in respiratory medicine.

## Author Contributions

All authors were involved in the development of the manuscript. MU designed the outline of this manuscript and co-wrote it with HW, AM, and FP. All authors have proofread the drafts and edited/approved the final manuscript.

### Conflict of Interest Statement

MU and AM are employees of AstraZeneca and hold shares in the company. AstraZeneca provided the AZD5069 compound for the exploratory research in this manuscript. The remaining authors declare that the research was conducted in the absence of any commercial or financial relationships that could be construed as a potential conflict of interest.
